# Human airway epithelium controls *Pseudomonas aeruginosa* infection via inducible nitric oxide synthase

**DOI:** 10.3389/fimmu.2024.1508727

**Published:** 2024-12-03

**Authors:** Philipp Grubwieser, Nina Böck, Erika Kvalem Soto, Richard Hilbe, Patrizia Moser, Markus Seifert, Stefanie Dichtl, Miriam Alisa Govrins, Wilfried Posch, Thomas Sonnweber, Manfred Nairz, Igor Theurl, Zlatko Trajanoski, Günter Weiss

**Affiliations:** ^1^ Department of Internal Medicine II, Infectious Diseases, Immunology, Rheumatology, Medical University of Innsbruck, Innsbruck, Austria; ^2^ Institute of Hygiene and Medical Microbiology, Medical University of Innsbruck, Innsbruck, Austria; ^3^ Biocenter, Institute of Bioinformatics, Medical University of Innsbruck, Innsbruck, Austria; ^4^ INNPATH, Innsbruck Medical University Hospital, Innsbruck, Austria; ^5^ Christian Doppler Laboratory for Iron Metabolism and Anemia Research, Medical University of Innsbruck, Innsbruck, Austria

**Keywords:** airway organoids, *Pseudomonas aeruginosa*, iNOS, airway epithelia, innate immunity

## Abstract

**Introduction:**

Airway epithelial cells play a central role in the innate immune response to invading bacteria, yet adequate human infection models are lacking.

**Methods:**

We utilized mucociliary-differentiated human airway organoids with direct access to the apical side of epithelial cells to model the initial phase of *Pseudomonas aeruginosa* respiratory tract infection.

**Results:**

Immunofluorescence of infected organoids revealed that *Pseudomonas aeruginosa* invades the epithelial barrier and subsequently proliferates within the epithelial space. RNA sequencing analysis demonstrated that *Pseudomonas* infection stimulated innate antimicrobial immune responses, but specifically enhanced the expression of genes of the nitric oxide metabolic pathway. We demonstrated that activation of inducible nitric oxide synthase (iNOS) in airway organoids exposed bacteria to nitrosative stress, effectively inhibiting intra-epithelial pathogen proliferation. Pharmacological inhibition of iNOS resulted in expansion of bacterial proliferation whereas a NO producing drug reduced bacterial numbers. iNOS expression was mainly localized to ciliated epithelial cells of infected airway organoids, which was confirmed in primary human lung tissue during *Pseudomonas* pneumonia.

**Discussion:**

Our findings highlight the critical role of epithelial-derived iNOS in host defence against *Pseudomonas aeruginosa* infection. Furthermore, we describe a human tissue model that accurately mimics the airway epithelium, providing a valuable framework for systemically studying host-pathogen interactions in respiratory infections.

## 1 Introduction

Bacterial lower respiratory tract infections remain a major public health concern, causing over 2 million deaths worldwide annually (1). Substantial progress has been made in understanding host responses to these infections. Animal and *in-vitro* models have underscored the importance of innate immune cells, such as macrophages, in frontline defence against pulmonary infections (2, 3). However, the role of the respiratory epithelium in early defence against bacterial pathogens is increasingly recognized (4). Positioned at the interface between the external environment and the internal milieu, the respiratory epithelium is the primary site of contact with invading bacteria, suggesting its active participation in early innate immune defence (5).

*Pseudomonas aeruginosa* (PA) is a gram-negative opportunistic bacterium and a growing health concern due to its pathogenicity and antibiotic resistance (6). PA lung infections are common and associated with high mortality rates, greatly impacting patients with cystic fibrosis (CF),chronic obstructive pulmonary disease (COPD) as well as patients with nosocomial pneumonia (7, 8).

During infection of the respiratory tract, PA closely interacts with epithelial cells and can adopt an intracellular lifecycle (9). PA can invade and actively replicate inside various epithelial cell types, including bronchial epithelia (10–12). Consequently, it has been hypothesized that epithelial internalization is the prerequisite for invasion, facilitating dissemination of the bacterium to the bloodstream and distant organs, while allowing the pathogen to evade innate immune cells, thus promoting bacterial persistence (13–15).

Although the airway epithelium initiates an inflammatory response and secretes antimicrobial effector molecules after pathogen detection, little is known about its direct antimicrobial responses. Only recently, we showed that airway epithelial cells differentially regulate nutrient trafficking in response to intra- or extracellular bacteria, thereby affecting pathogen multiplication (16).

It has been hypothesized that the production of reactive oxygen- and nitrogen species (ROS/RNS) by airway epithelial cells facilitates intracellular pathogen killing (5). Indeed, the human respiratory epithelium expresses nitric oxide synthases (NOS) (17, 18). In mice, the expression of the inducible isoform iNOS, mainly by monocytic cells including macrophages, is induced by inflammatory stimuli, such as bacterial lipopolysaccharide (LPS) or cytokines, such as interferon-gamma, tumor-necrosis factor-alpha, and interleukin- (IL-) 17 (19, 20). However, limited information is available on iNOS function in human infection models, particularly regarding its potential role within the human respiratory epithelium (21).

Despite significant progress, the multistage and cell specific mechanisms of host responses to bacterial invasion in the lung remain poorly understood. Herein we apply a novel infection model, in which differentiated human airway organoids (AOs) are challenged with the opportunistic pathogen *Pseudomonas aeruginosa*. In direct suspension culture, apical-out organoids are exposed to viable bacteria, closely mimicking the *in-vivo* situation as bacteria interact with the epithelial layer at the correct spatial localization. This enables monitoring of the bacterial entry and intracellular fate, and in parallel allows for comprehensive analysis of host epithelial cell responses to infection.

## 2 Material and methods

### 2.1 Organoid and bacteria culture

Patient derived human airway organoids were acquired from Foundation Hubrecht Organoid Biobank (www.hubrechtorganoidbiobank.org) and cultured as described by Sachs et al. (22). Apical-out polarity switch and mucociliary differentiation was performed as described by Co et al. (23) and Zhou et al. (24), respectively.

*Pseudomonas aeruginosa*, strain P14 as well as strain P14 stably expressing GFP (PA-GFP), were a kind gift of Dirk Bumann, Biozentrum Basel, Switzerland. This reference strain is characterized by its similarity to patient isolates in terms of virulence and thus is the preferred model for infection studies with virulent PA (25). Detailed information is provided in the **Supplementary Methods**.

### 2.2 Organoid infection

Fully differentiated, apical-out human airway organoids were washed two times with PBS, and seeded in equal density in proximal differentiation medium without antibiotics and hydrocortisone into 12-well plates treated with anti-adherence solution. On the next day, PA from the mid-logarithmic growth phase was added to organoids at a final concentration of 25*10^6^/ml for 3h. Bacterial outgrowth in the medium is then prevented by washing thrice in PBS containing gentamicin (25µg/ml) and adding fresh medium containing gentamicin (8µg/ml) for further incubation. To minimize the use of gentamicin and thus off-target effects, we determined the minimal inhibitory concentration (MIC) of gentamicin with conventional microbiological methods (Etest, MIC: 1µg/ml, **Supplementary Figure 2A**) and in experiment-specific conditions (growth in ALI or LB medium; **Supplementary Figure 2B**). This antibiotic is not able to penetrate cell membranes, and thus only acts in the extracellular space (26). Thus, bacteria, which have invaded the epithelial formation, are not exposed to this antibiotic and remain viable inside epithelial cells. During this gentamicin-protected phase, organoids and intra-organoid bacteria further interact for up to 24 hours, enabling analysis of intra-organoid bacterial numbers and organoid innate immune responses at various time intervals. To quantify intra-organoid bacteria, AOs were washed thrice in PBS, and subsequently lysed in 0.5% sodium deoxycholic acid (Sigma-Aldrich). Organoid lysates, containing viable bacteria, were plated immediately on LB plates, and colony-forming units (CFUs) were quantified after overnight incubation. Where indicated, Organoids were treated with 100µM of the NO-donor NOC-18 (MedchemExpress, HY-136278) or 50µM of the iNOS inhibitor L-NIL (MedchemExpress, HY- 12116) during the active and gentamicin-protected infection phase.

### 2.3 Statistical analysis

RNA sequencing analysis is described in detail in the **Supplementary Methods** section. Statistical analysis of CFU and qPCR data was performed in GraphPad Prism (9.4.1). For pairwise comparisons, an unpaired student t-test was used. ANOVA with Sidaks *post-hoc* test was used for multiple group comparisons. Data was log-transformed as appropriate. A p-value of <0.05 was used as the significance threshold. Additional methods can be found in the **Supplementary Material**.

## 3 Results

### 3.1 *Pseudomonas aeruginosa* infects differentiated human airway organoids and resides in the intracellular space

**Figure 1:**
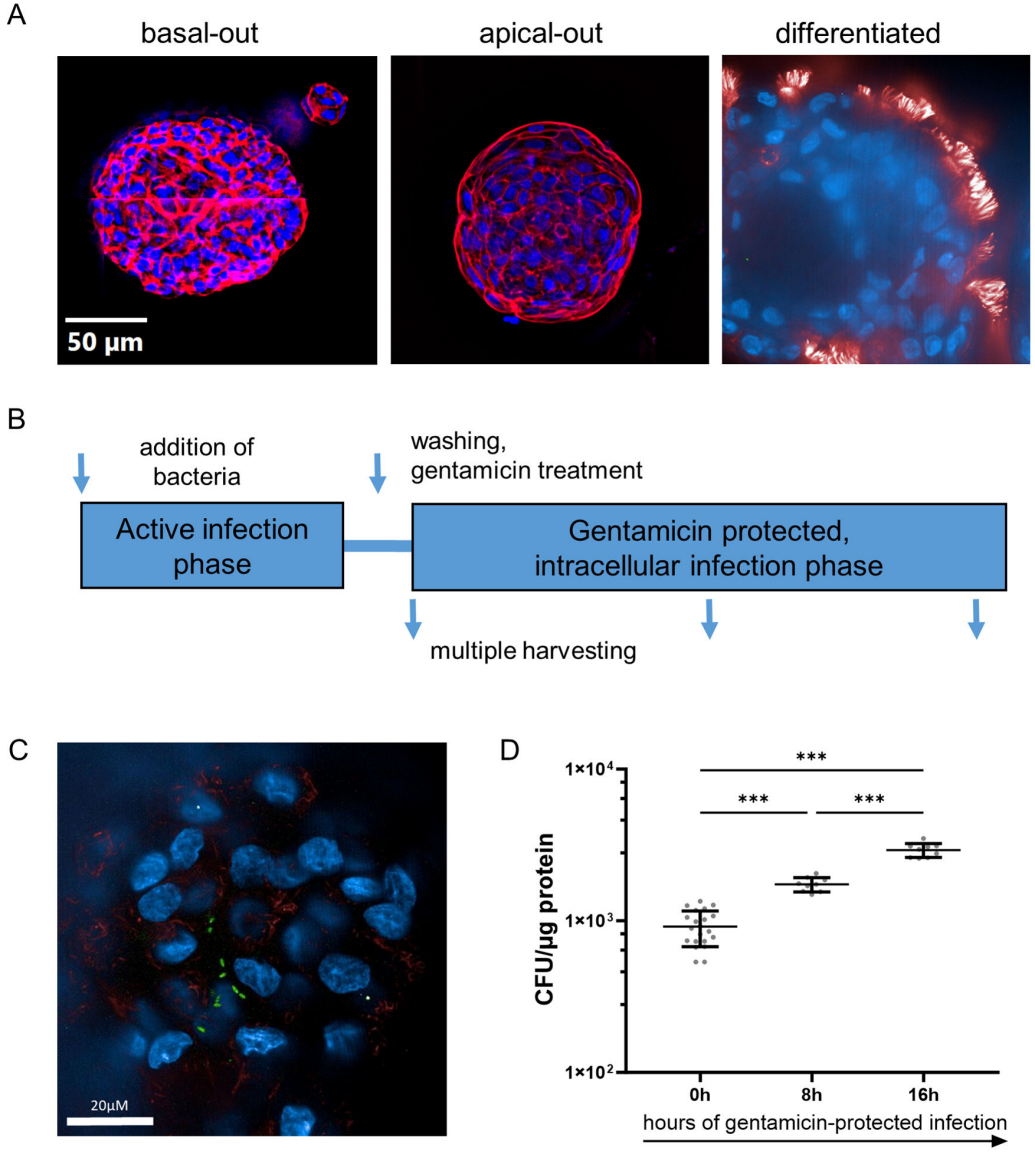
*Pseudomonas aeruginosa* infects differentiated human airway organoids and resides in the intracellular space. **(A)** Immune fluorescence imaging of human airway organoids in basal-out configuration (left panel) and apical-out configuration (middle panel). Representative epifluorescence images of organoids stained for actin (red) and nuclei (blue), mounted on slides. Terminally differentiated organoid (right panel). Representative confocal image, stained for apically located cilia (red) and nuclei (blue). **(B)** Schematic representation of the organoid infection model used herein. **(C)** Confocal immune fluorescence imaging revealing intra-organoid bacteria (PA-GFP, green rods) in infected organoids after 3h of active infection and removal of extracellular bacteria. **(D)** Quantification of intracellular bacteria during the gentamicin-protected course of infection. Infected organoids were lysed at indicated time intervals, and lysates were plated onto LB-agar plates for CFU quantification. Data from three independent experiments are shown as a scatter plot with mean ± SD. *** denotes p < 0.001 for ANOVA with *post hoc* statistical testing.

To investigate the epithelium-pathogen interaction, we first generated apical-out airway organoids. To this end, we treated organoids with EDTA for complete matrix degradation and cultured them in suspension, resulting in apical-out airway organoids (AOAOs, **Figure 1A**). After 16 days of culture in the differentiation medium, AOAOs present an abundance of ciliated cells at the apical side, considered to be the hallmark of terminal mucociliary differentiation. During the differentiation phase, markers of ciliated (FOXJ1) and goblet (MUC5AC) cells increased significantly, whereas club cells (SCGB1A1) decreased, and basal cells (P63) remained constant (**Supplementary Figure 1**).

AOAOs were then challenged with the bacterial pathogen PA in a gentamicin-protected infection model (**Figure 1B**): viable bacteria are directly added to the antibiotic-free cell culture medium, after which AOs and bacteria directly interact for 3h. Subsequently, to prevent bacterial overgrowth, AOs are washed and treated with gentamicin, which eliminates bacteria in the extracellular space but does not affect intracellular bacteria which have invaded the organoid. Infected AOs are then further incubated in a gentamicin-containing medium and harvested at multiple time intervals for analysis.

We first applied immunofluorescence of organoids infected with PA expressing GFP (PA-GFP), harvested after the active infection phase and after the removal of extracellular bacteria. Interestingly, confocal imaging revealed bacteria (green, rod-shaped) inside the epithelial tissue (**Figure 1C**), showing that PA has invaded the epithelial barrier. Subsequently, to quantify the number of viable intracellular bacteria, the organoid lysate was plated at multiple time intervals of the gentamicin-protected phase (**Figure 1D**). Viable PA bacteria were recovered directly after the active infection phase (0h), indicating the number of bacteria that have invaded AOs. The number of intra-organoid bacteria subsequently increased, with peak bacterial burden at 16h, suggesting intra-organoid bacterial multiplication.

### 3.2 Pro-inflammatory signaling shapes organoid responses to infection

**Figure 2:**
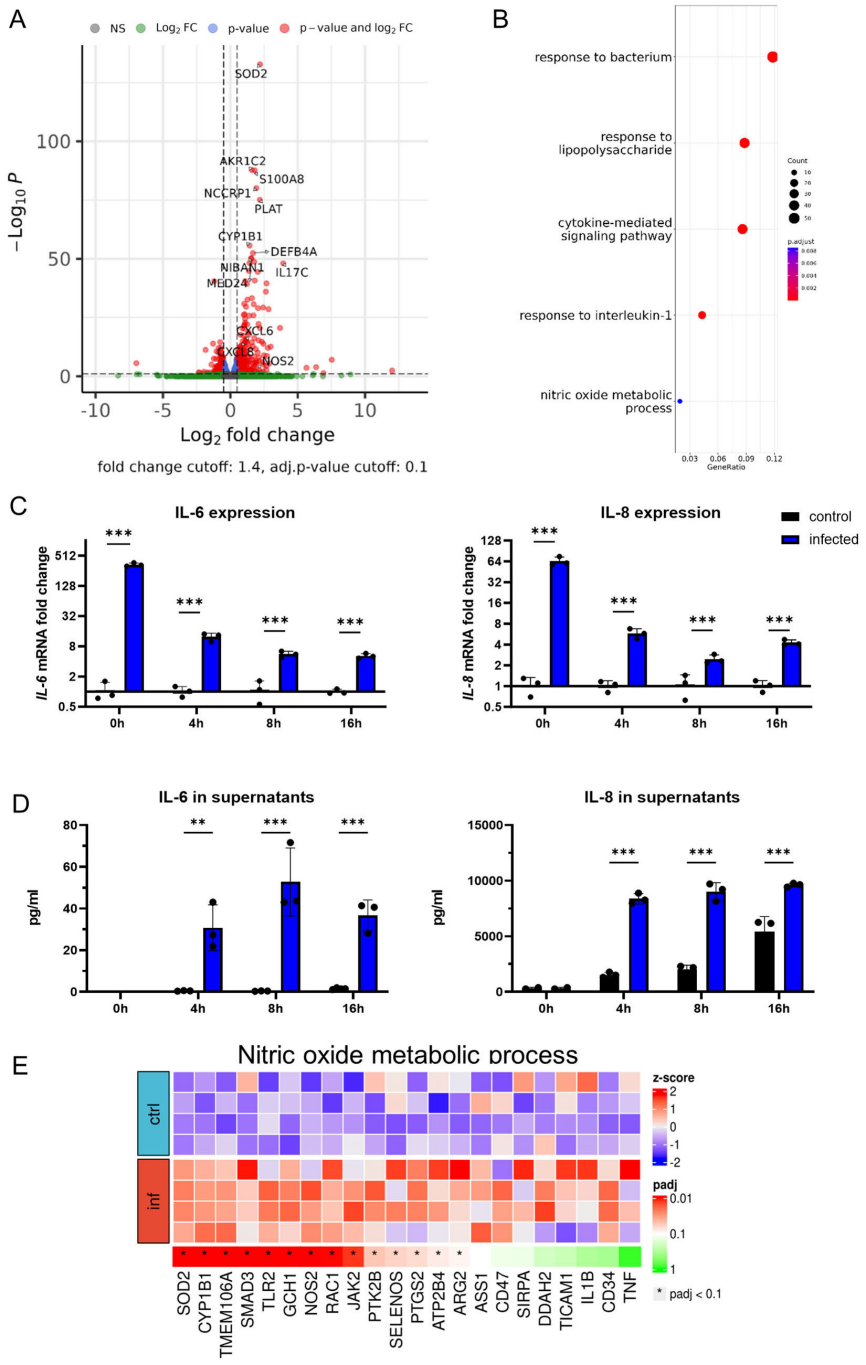
Pro-inflammatory signaling shapes organoid responses to infection. **(A)** Volcano plot showing differentially expressed genes of infected human airway organoids in comparison to uninfected controls after 4h of gentamicin-protected infection. **(B)** Gene-set analysis with over-representation test (ORA) showing Gene ontology biological processes. **(C)** Differential IL-6 (left panel) and IL-8 (right panel) mRNA expression in infected organoids. Organoids were harvested at indicated time intervals of gentamicin-protected infection. Data is shown as mean ± SD of a triplicate experiment. **(D)** Levels of the inflammatory cytokines IL-6 (left panel) and IL-8 (right panel) in supernatants of infected organoids. Supernatants were collected at indicated time intervals of gentamicin-protected infection. Data is shown as mean ± SD of a triplicate experiment. **(E)** Heat map graphical representation of differentially expressed genes in infected versus uninfected organoids. The genes (X axis) are derived from Gene ontology nitric oxide metabolic process (GO:0046209) set of genes. The gene expression is indicated by z-score. ** denotes p < 0.01, *** denotes p < 0.001 for ANOVA with *post-hoc* statistical testing.

To shed light on organoid responses to infection, bulk RNA sequencing of infected and uninfected organoids was performed. Direct comparison revealed major differences in the transcriptome of infected organoids compared to uninfected controls (**Figures 2A, B**). Principal-component analysis (PCA) demonstrated close similarities among samples belonging to the same treatment group after batch effect correction (**Supplementary Figure 3**). Infection with PA induced the upregulation of 343 genes and downregulation of 182 genes in AOs, with a significant increase in the expression of genes associated with the inflammatory response, such as the pro-inflammatory cytokine IL-17C (Volcano plot, **Figure 2A**; **Supplementary Table 2**). The ORA revealed, that these changes reflect pathways involved in cellular responses to bacteria and lipopolysaccharides (LPS), mainly including a pro-inflammatory cytokine signature (IL1-B, TNFA, IL-8) and production of antimicrobial peptides (DEFB, S100A8, S100A9). To confirm if those alterations are also found by sequential analysis of mRNA and protein expression of such genes, we studied IL-6 and IL-8 expression via qPCR and ELISA over time (**Figures 2C, D**). The transcriptional induction of both cytokines was immediately increased after the initiation of the infection phase and significantly elevated throughout the gentamicin-protected infection phase, being in line with the RNA-sequencing data. Consistent with previous findings, uninfected AOs exhibited basal secretion of IL-8 (27). Interestingly, aside from the expected inflammatory response, differential regulation of metabolic pathways, specifically the nitric oxide (NO) metabolic pathway (GO:0046209) was evident (**Figure 2E**). This pathway contains several genes associated with nitric oxide production, including iNOS (NOS2), which was significantly induced in infected organoids compared to uninfected controls. Together, these results indicate that human airway organoids exposed to viable bacteria induce multiple inflammatory and anti-microbial pathways, secrete inflammatory cytokines and upregulate genes that are associated with NO production.

### 3.3 Human airway organoids induce iNOS in response to infection with *Pseudomonas aeruginosa*

**Figure 3:**
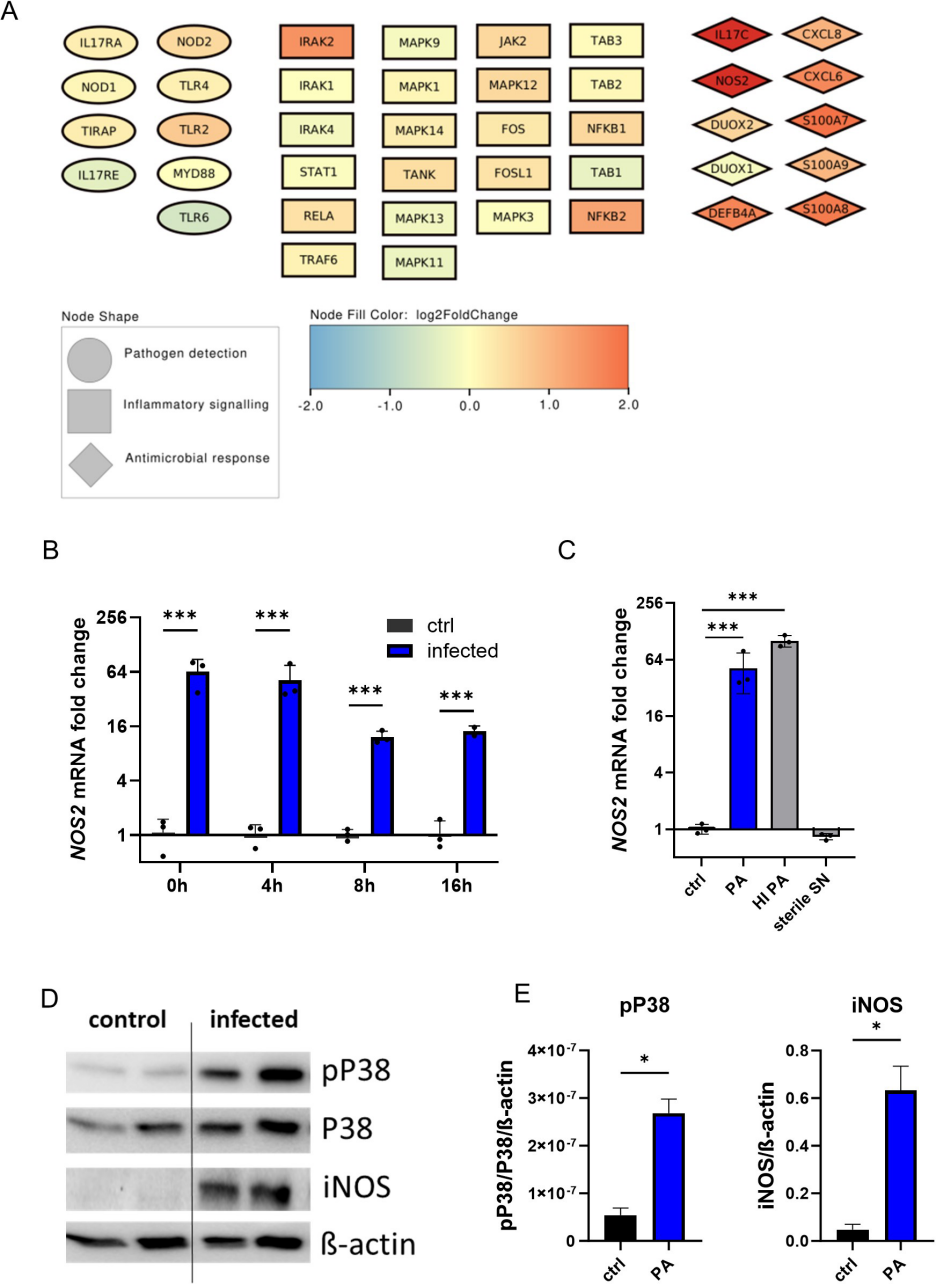
Human airway organoids induce iNOS in response to infection with Pseudomonas aeruginosa. **(A)** Graphical representation of infection-associated induced genes as nodes. Pathogen-detection-associated genes are shown as ellipses, intracellular inflammatory signalling genes are shown as squares, and antimicrobial effectors are depicted as diamonds. The colour of the nodes indicates the log2-fold change from the differential expression analysis of infected human airway organoids compared to uninfected controls **(B)** Differential NOS2 mRNA expression in infected organoids. Organoids were harvested at indicated time intervals of gentamicin-protected infection. Data is shown as mean ± SD of three independent experiments. **(C)** Differential NOS2 mRNA expression at the 4h time interval in infected organoids and organoids exposed to heat-inactivated (HI) bacteria or treated with sterile-filtrated bacterial supernatant (SN). Organoids were harvested at indicated time intervals of gentamicin protected infection. Data is shown as mean ± SD of three independent experiments. **(D)** Western blot of the MAPK subunit p38, phosphorylated p38, and iNOS protein of infected organoids and uninfected controls harvested directly after the active infection phase (0h-time interval), shown in duplicates. **(E)** Densitometric quantification of the Western blots targets iNOS and phosphorylated p38. * denotes p < 0.05 for statistical testing with a two-sided unpaired t-test, *** denotes p < 0.001 for ANOVA with post-hoc statistical testing.

Next, to further examine organoid iNOS induction in response to infection, we evaluated the involved regulatory networks. To accomplish this, bulk RNA sequencing data was analysed for pathogen detection and subsequent activation of inflammatory signalling pathways, which finally led to antimicrobial effector induction (**Figure 3A**). In all entities, a significant positive regulation pattern in infected organoids was evident, including a prominent induction of iNOS. We confirmed the transcriptional upregulation of iNOS by qPCR at multiple time-intervals of the gentamicin-protected infection phase (**Figure 3B**). A striking increase in NOS2 mRNA levels is visible directly after the end of the active infection phase (3h of bacterial exposure), with a consecutive upregulation thereafter. Interestingly, not only infection with viable PA but also treatment with heat-inactivated bacteria (HI PA) led to a significant increase of NOS2 mRNA levels (**Figure 3C**). In contrast, treatment with the sterile bacterial supernatant (SN), which contains soluble bacterial products including toxins but no viable bacteria, did not result in iNOS induction. Notably, the induction of iNOS in infected organoids was evident at the protein level (**Figures 3D, E**). Induction of iNOS was paralleled by increased phosphorylation of p38, indicating increased mitogen-activated protein kinase (MAPK) activation. This data suggests, that the host-pathogen interaction during active PA infection leads to the activation of pro-inflammatory pathways in airway epithelial cells including iNOS formation at the transcriptional and translational level.

### 3.4 iNOS is induced in ciliated cells of infected airway organoids and human bronchial epithelia during *Pseudomonas aeruginosa* pneumonia

**Figure 4:**
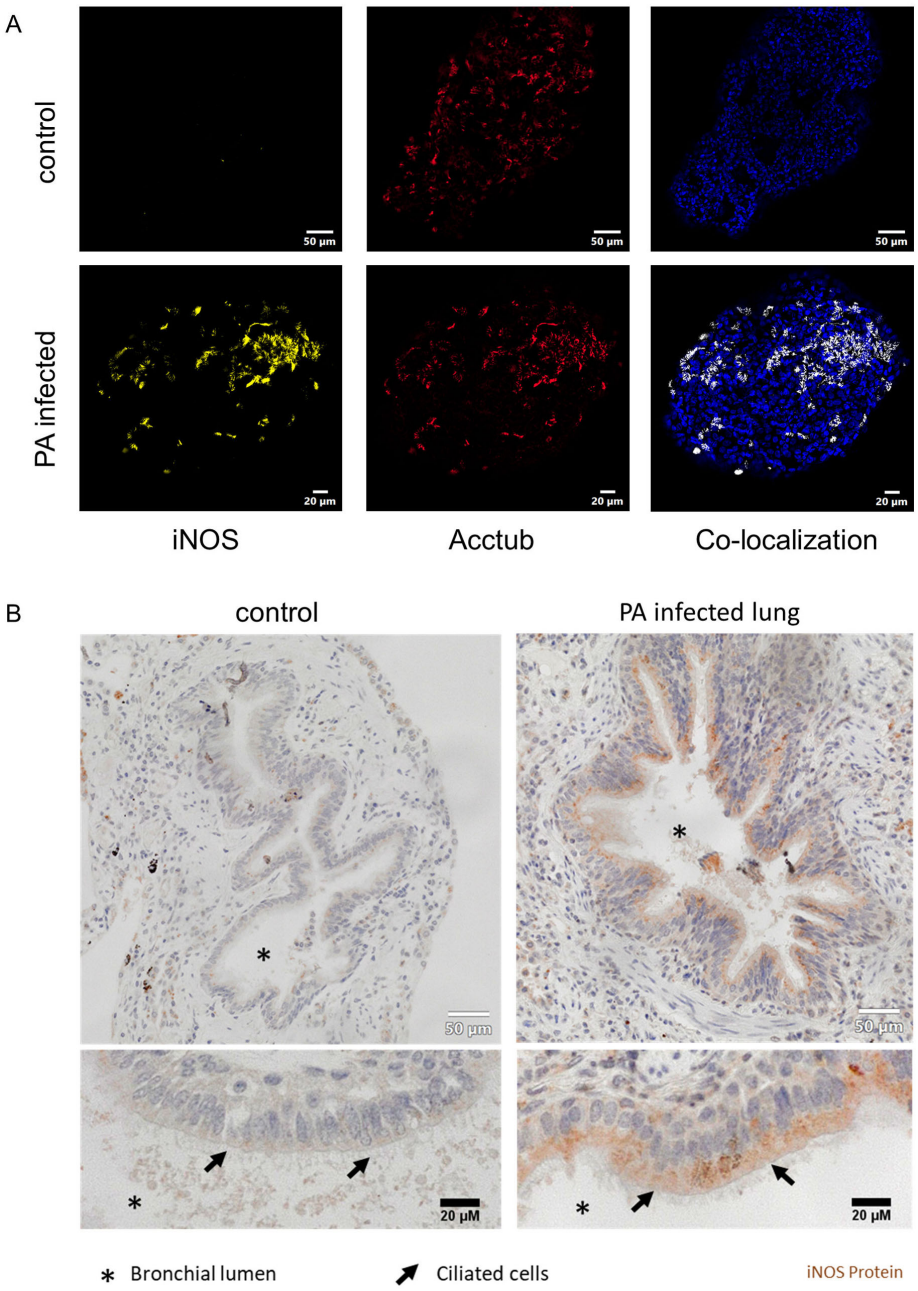
iNOS is induced in ciliated cells of infected airway organoids and human bronchial epithelia during *Pseudomonas aeruginosa* pneumonia. **(A)** Immune fluorescence imaging of differentiated human airway organoids, infected with PA14 (lower panels). Representative epifluorescence images of organoids stained for iNOS (yellow), ciliated cells (red) and nuclei (blue). Co-localization of iNOS and ciliated cells is depicted in white (right panel). **(B)** Histological analysis of human lung samples stained for iNOS protein obtained from a non-infected lung (left panel) and a lung with confirmed *Pseudomonas aeruginosa* pneumonia (right panel).

Next, we applied immunofluorescence imaging to reveal cellular localization of iNOS-protein expression in infected organoids. After the active infection, organoids were stained for ciliated cells (acetylated tubulin) and iNOS protein. Imaging revealed induction of iNOS protein in infected organoids but not in uninfected controls (**Figure 4A**). Furthermore, regions with high iNOS expression in infected AOs structurally resembled ciliated cells. Indeed, co-staining with acetylated tubulin, a specific marker for ciliated cells, revealed near congruent expression of iNOS protein (**Figure 4A**). Finally, iNOS expression was investigated in human lung tissue specimen (**Figure 4B**). Immunohistochemistry staining confirmed iNOS protein presence in the bronchial epithelium in a patient infected with PA, specifically in ciliated cells of the bronchial region. Compared to this, no obvious iNOS expression was found in a non-infected control lung sample. These results indicate, that iNOS is induced in human respiratory epithelium upon pathogen contact, specifically in ciliated cells. Corroborating our findings of the organoid infection model, the iNOS protein expression showed a similar pattern in histological analysis of human lung specimens.

### 3.5 Human airway organoids expose *Pseudomonas aeruginosa* to nitrosative stress

**Figure 5:**
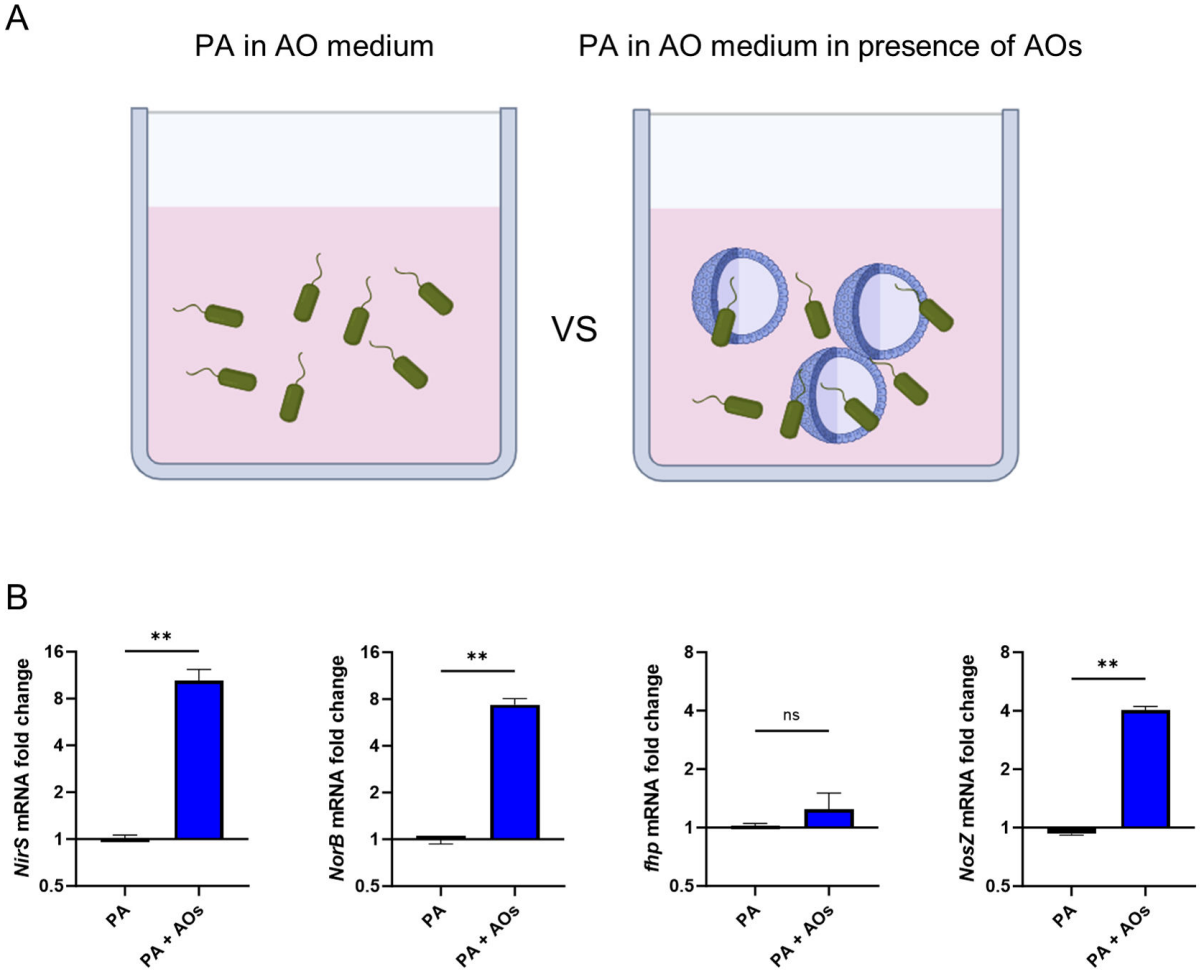
Human airway organoids expose *Pseudomonas aeruginosa* to nitrosative stress. **(A)** Graphical illustration of the experimental setup: PA were either incubated for 3h in antibiotic-free AO medium alone or in the presence of AOs. After this, mRNA was extracted. **(B)** Differential mRNA expression of bacterial genes associated with denitrification, in PA versus PA exposed to human AOs. Data is shown as mean ± SD of three independent experiments. ns denotes not significant, ** denotes p < 0.01 for a two-sided, unpaired t-test.

After confirming the expression of iNOS protein in infected AOs, we next assessed the impact of iNOS expression on bacterial genes associated with response to nitrosative stress exerted by host formation of NO. For this, PA bacteria were either incubated in AO medium alone or in the presence of AOs (graphical representation **Figure 5A**). After 3h of incubation, mRNA was extracted and expression of bacterial genes associated with NO detoxification, namely nitrite reductase (NirS), nitric oxide reductase (NorCB), nitrous oxide reductase (NosZ), flavohemoglobin (fhp)) were analysed (**Figure 5B**) (28). In bacteria exposed to AOs, several genes for detoxification of nitric-oxide stress were significantly elevated, indicating that direct interaction with human AOs leads to the induction of nitrosative stress in bacteria and subsequently to expression of bacterial NO detoxification enzymes.

### 3.6 Human airway organoids control intracellular *Pseudomonas aeruginosa* growth *via* iNOS

**Figure 6:**
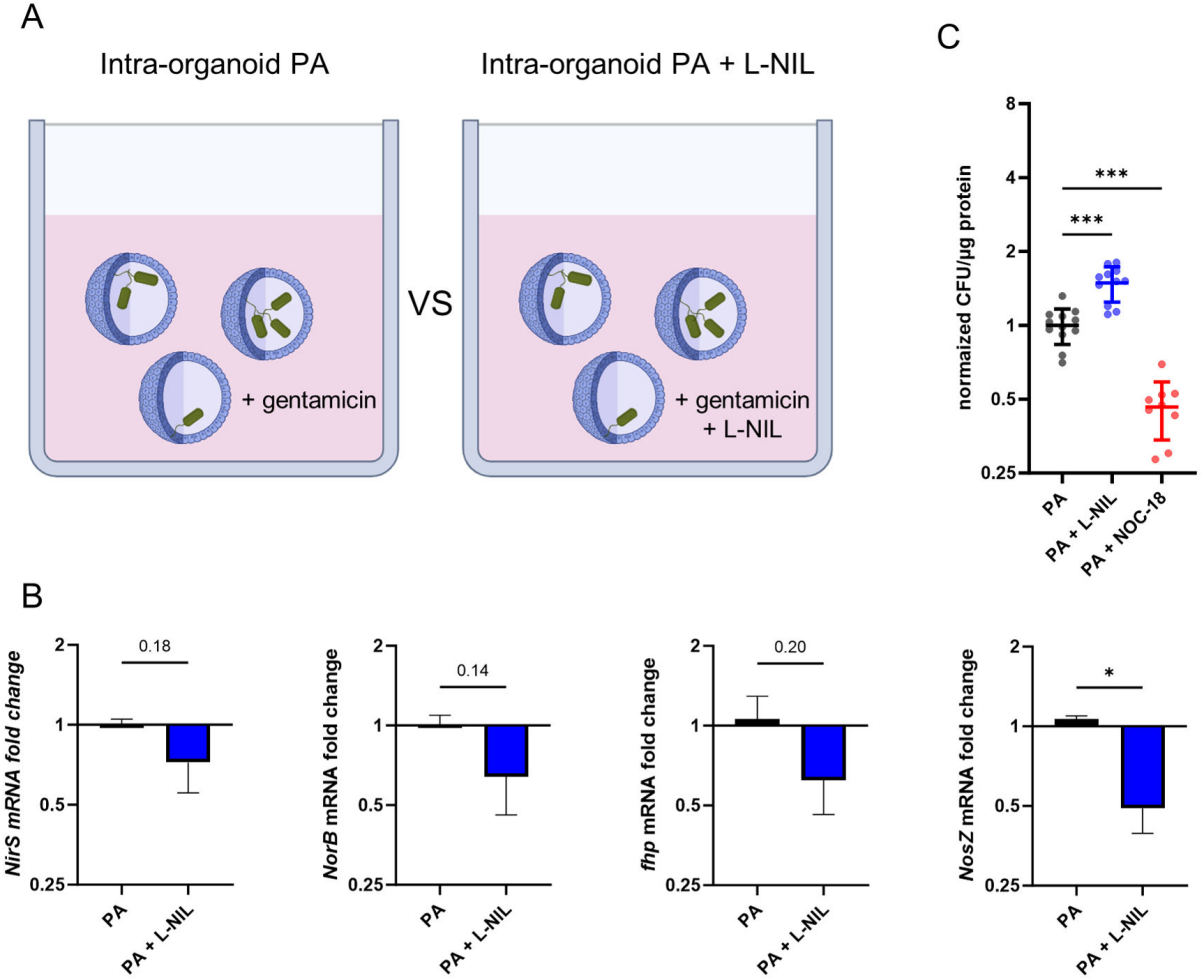
Human airway organoids control intracellular *Pseudomonas aeruginosa* growth *via* iNOS. **(A)** Graphical illustration of the experimental setup: Infected AOs were incubated with or without the specific iNOS inhibitor L-NIL during the 16h gentamicin-protected infection phase. Afterwards, mRNA was extracted. **(B)** Differential mRNA expression of bacterial genes associated with denitrification, in PA infecting AOs versus PA infection of AOs in the presence of the iNOS inhibitor L-NIL after 16h of gentamicin protected infection. Data is shown as mean ± SD of three independent experiments. **(C)** Quantification of intracellular bacteria after 24h of gentamicin-protected infection of AOs treated with 50µM iNOS inhibitor L-NIL, 100µM NO-donor NOC-18 or left untreated. Infected organoids were lysed, and lysates were plated onto LB-agar plates for CFU quantification. Data from three independent experiments performed in triplicates are shown as a scatter plot with mean ± SD, normalized to infection control (PA). * denotes p < 0.05 for unpaired t-test, *** denotes p < 0.001 for ANOVA with *post-hoc* statistical testing.

To determine the functional relevance of epithelial iNOS expression for the control of intraepithelial bacterial multiplication, we applied the specific iNOS inhibitor L-NIL in our AO infection model (illustration **Figure 6A**). To this aim, AOs were infected with PA and treated with 50µM L-NIL during the active infection and gentamicin-protected infection phase, or left untreated. At the peak of bacterial intra-organoid pathogen burden (16h), total RNA was extracted and expression of bacterial genes associated with denitrification (*NirS, NorB, fhp, NosZ*) were analysed (**Figure 6B**). In intra-organoid bacteria from AOs treated with the iNOS inhibitor L-NIL, expression of denitrification genes (*NirS, NorB, fhp, NosZ*) was generally lower compared to bacteria exposed to AOs under standard conditions. A statistically significant reduction was only observed for the nitrous oxide reductase (*NosZ*). Next, intra-organoid bacteria were quantified after the gentamicin-protected infection phase in AOs treated with either the iNOS inhibitor L-NIL or the NO-donor NOC-18 (**Figure 6C**). Underlining the role of iNOS activation in pathogen control, recovered CFUs from AOs treated with L-NIL were significantly increased. Accordingly, bacterial numbers recovered from AOs treated with the NO forming drug NOC-18 were significantly decreased. In sum, this data demonstrates that human airway organoids infected with PA control intra-epithelial bacterial growth at least in part by induction of the iNOS pathway in epithelial cells.

## 4 Discussion

To date, a bottleneck in respiratory infection research is the sparse offering of physiologically relevant *in-vitro* or *ex-vivo* models to study bacterial interactions with human airway epithelial cells. Here, we developed an *ex-vivo* human model and demonstrate that human mucociliary differentiated bronchiolar airway organoids recapitulate the early steps of *Pseudomonas aeruginosa* respiratory tract infection. AOs recognize invading pathogens and initiate innate immune responses, as indicated by induction of multiple inflammatory and anti-microbial pathways, which is consistent with previous studies using *Pseudomonas*-epithelial infection models (29, 30). Furthermore, suppressive regulators of innate immunity, like TRIM29 or IL-19, showed a positive regulation pattern in infected AOs. TRIM29 has been reported to be highly expressed by airway epithelial cells and is modulating innate immune responses (31, 32). Interestingly, increased presence of TRIM29 promotes endoplasmic reticulum stress and production of reactive species, potentially exposing an intracellular pathogen to oxidative stress (33). Considering this dual role in both innate immune modulation and stress response, epithelial-derived TRIM29 potentially impacts PA respiratory tract infection.

In this work, we provide the first evidence of the airway epithelial iNOS axis in controlling intra-organoid bacterial growth. Although iNOS activity is a well-established antimicrobial effector molecule in murine infection models, the role of iNOS-derived antimicrobial RNS in humans remained less clear (20, 34). Most human *ex-vivo* infection models employing innate immune cells, such as macrophages, failed to show induction of iNOS during infection or sufficient production of antimicrobial iNOS-derived RNS species. Several reasons have been postulated, including epigenetic silencing of the respective promotor or the artificial environment during *ex-vivo* experiments (35, 36). Nevertheless, induction of iNOS following infection has been demonstrated for specific infections *in-vivo*, including tuberculosis (37).

In respiratory infections, it has been proposed that iNOS may not originate from classical innate immune cells, such as macrophages, but rather from airway epithelial cells (17, 18). Accordingly, dysfunctional airway epithelium, for instance in CF or COPD patients, critically alters host defence functions (38). In a cellular co-culture model, CF bronchial epithelial cells failed to induce iNOS in response to neutrophilic infiltration (39). In line with this observation, lower epithelial iNOS expression was shown in CF subjects as compared to controls (40). Nonetheless, NO formation in a CF epithelial cell-line model resulted in decreased PA adherence and improved the elimination of internalized bacteria (21). Together, these findings suggest that the reduced functionality of epithelial iNOS in CF patients significantly contributes in PA colonization and infection. Additionally, the use of inhaled corticosteroids in COPD patients is associated with an increased pneumonia rate, potentially due to decreased epithelial NO production (41).

To determine the role of epithelial iNOS production in immune defence against PA in our airway organoid model, we used the specific pharmacological iNOS inhibitor L-NIL or the NO-forming drug NOC-18. We observed enhanced clearance of PA upon addition of NOC-18, whereas treatment with L-NIL increased bacterial numbers in our organoid infection model. Together, these findings confirmed that upregulation of iNOS activity upon PA infection plays critical part in epithelial infection control. The upregulation of iNOS in epithelial cells by both viable and heat-inactivated PA suggests that bacterial cell wall components, as well as interaction with intracellular PAMPs induce iNOS expression.

An improved understanding of innate immune responses mediated by airway epithelial cells is pivotal to effectively combat early infection and impede PA persistence, especially given the rising burden of anti-microbial resistance. This may contribute to the development of adjunct therapeutic concepts by modulating specific innate immune pathways, including the stimulation of iNOS-mediated antimicrobial effector mechanisms, in order to improve infection outcomes.

## Data Availability

The original contributions presented in the study are publicly available. This data can be found here: European Nucleotide Archive, accession number: PRJEB82833.
