# Supplementary material for: Human airway epithelium controls Pseudomonas aeruginosa infection via inducible nitric oxide synthase
Source: Front Immunol. 2024 Dec 3;15:1508727. doi: 10.3389/fimmu.2024.1508727 (PMC11649544; doi:10.3389/fimmu.2024.1508727)
Supplement: Supplementary file 1 [file DataSheet1.docx]

Supplementary Material

# Supplementary Methods

## Organoid and bacteria culture

Human airway organoids were acquired from Foundation Hubrecht Organoid Biobank (www.hubrechtorganoidbiobank.org). Organoids were expanded in 30 µL Geltrex™ LDEV-Free Reduced Growth Factor Basement Membrane Matrix (Thermo Scientific, Cat#A1413202) embedded in airway organoid expansion medium as described by Sachs et al. (1) in 6-well plates. Organoids were split via TrypLE™ Express Enzyme (Invitrogen, Cat#12604013) matrix degradation and re-embedding in Geltrex every 4-6 days. For experiments, organoids were harvested and epithelial polarization was changed from basal-out to apical-out configuration via EDTA-treatment (1h, 4°C) as described by Co et al. (2). After EDTA-treatment, organoids were kept in suspension culture in 6-well plates treated with Anti-Adherence Rinsing Solution (Stem Cell, Cat#07010) in expansion medium for two more days. Subsequently, the medium was changed to a proximal differentiation medium, consisting of complete Pneumacult ALI medium (Stemcell) supplemented with 2IU/ml Heparin, 1µM Hydrocortisone (Stemcell), 10µM DAPT (Stemmcell) and Penicillin-Streptomycin first described by Zhou et al. (3) for 16 days.

*Pseudomonas aeruginosa* (PA), strain P14 as well as strain P14 stably expressing GFP (PA-GFP) were a kind gift of Dirk Bumann, Biozentrum Basel, Switzerland. This reference strain is characterized by its similarity to patient isolates in terms of virulence and thus is the preferred model for infection studies with virulent *Pseudomonas aeruginosa* (4). Bacteria were grown in overnight cultures in LB-medium at 37°C under constant shaking (200rpm). For infection experiments, a fresh culture was prepared from the overnight culture and grown until OD_600_ (optical density at 600nm) 0.5, which corresponds to the mid-logarithmic growth phase. For experiments with heat-inactivated bacteria, PA was incubated at 70°C for 20 minutes. To confirm the absence of viable bacteria, an aliquot of heat inactivated PA was plated onto LB agar. For experiments with sterile bacterial supernatant, an overnight culture of PA was centrifuged at 4600G for 10 minutes, and subsequently the supernatant was sterile filtered through a 0.2 µM filter.

## RNA Sequencing

FASTQ files from 16 samples from 2 different batches were processed with the nf-core RNA-seq pipeline version 3.10.1 [10.5281/zenodo.1400710]. In brief, reads were trimmed with TrimGalore v0.6.10 [10.5281/zenodo.5127899] and were afterwards aligned to the GRCh38 reference genome with GENCODE v38 annotation, using STAR 2.7.10b. Gene expression was quantified using Salmon v1.10.0 [5]. Thereafter, the gene counts tables were imported into R (v.4.2.3) for downstream analysis. Differential gene expression was performed between infected and control conditions using Bioconductor packages DESeq2 v1.38.3 [6]. To control for batch effect the biological replicate information was added to the DESeq2 design matrix. False-discovery-rates (FDR) were calculated using independent hypothesis weighting (IHW) as a multiple testing procedure v1.26 [7]. The filtering of the gene list was performed using threshold values: adjusted P adj.-value < 0.1 and |log2FC| ≥ 0.5. The result of the differential expression analysis was used as an input for the over-representation analysis (ORA). ORA was performed with the clusterProfiler package v.4.6.2 using the biological processes of the Gene Ontology (GO-BP) database and the Kyoto Encyclopedia of Genes and Genomes (KEGG) as input gene lists. Volcano plots were visualized using the R package EnhancedVolcano v1.16.0 using the before-mentioned thresholds. Heatmap plots were generated using ComplexHeatmap v2.14.0 where gene expression was represented using z-score.

## Immunohistochemistry

Histological lung specimens of a patient with *Pseudomonas aeruginosa* pneumonia and a healthy control patient, obtained during clinical routine, were used as approved by the Ethics committee at Medical University Innsbruck, Austria (study number 1046/2024). Immunohistochemistry staining of histological samples for iNOS protein (Abcam 3523) was performed according to standard protocols. The staining and imaging procedure for compared samples were performed on the same slide. For image acquisition, an Olympus BX61VS slide-scanner equipped with a 20x objective and the OlyVIA software were used.

## qPCR

The quantitative real-time PCR was carried out as described before [8]. Real-time PCR reactions were performed on QuantStudio 3 and 5 real-time PCR systems (Thermo Fisher Scientific). Gene expression was normalized using the ΔΔct method. Tubulin (TUB) and ornithine decarboxylase antizyme 1 (OAZ1) were used as reference transcripts for human genes, whereas 16s and RNA polymerase sigma factor (RpoD) were used for bacterial genes. TaqMan PCR primers used in this study are listed in Supplementary Table 1.

## Western Blot

Protein extraction, preparation, and Western blotting were performed as described previously [8]. Following antibodies were used: a rabbit iNOS antibody (1:500; Abcam, 3523), a rabbit p38 MAP kinase antibody (1:100, Cell Signaling, #9212), a rabbit phosphor-p38 MAP kinase antibody (1:1000, Cell Signaling, #4511), and a rabbit actin antibody (1:500, Sigma Cat# A2066). Following appropriate secondary antibodies were used: anti-rabbit (1:2000, Dako Cat# P0399), anti-mouse (1:4000, Dako Cat# P0447)

## ELISA

To measure cytokines in organoid supernatants, a Human IL-6/IL-8 High Sensitivity Magnetic Luminex® Performance Assay (LHSCM208, LHSCM206) was applied and measured in a Luminex® imager according to the manufacturer's instructions.

## Immunofluorescence imaging

Uninfected organoids or organoids infected with PA-GFP were washed and harvested at indicated the time points. Afterwards, organoids were fixed with 4% paraformaldehyde for 20 min and subsequently permeabilized by incubation in 0.5% saponin (Sigma-Aldrich) for 30 min. Subsequently, primary antibodies were used for overnight staining at 4°C: iNOS antibody (1:500, Abcam 3523), and acetylated tubulin antibody (1:100, Abcam, 218591). On the following day, organoids were stained with 4′,6-diamidino-2-phenylindole (1:10000, DAPI, BioLegend) and Phalloidin-iFlour 647 (1:1000, Abcam, 176759), and in the case of iNOS staining, a Goat anti-Rabbit secondary antibody (anti-rabbit 594 Invitrogen, A11012) for 2h at RT. Fluorescence microscopy was performed immediately after sample preparation using a VS120-S6 fluorescence microscope (Olympus) or the Operetta CLS System (PerkinElmer). Images were captured with a 20-x, 40-x, or 63-x objective using 387/440 nm, 485/525 nm, and 650/684 nm lasers and filters. To allow comparison of indicated samples, identical exposure times were used.

# Supplementary Figures

## Supplementary Figure 1


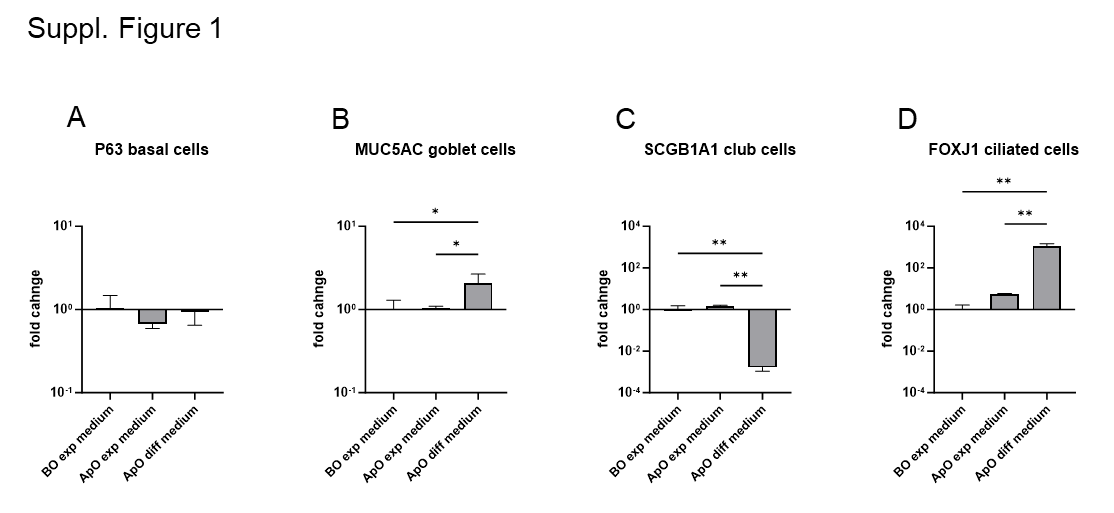


**Supplementary Figure 1.** Differential mRNA expression of cell-specific genes in AOs after 16 days of culture in either basal out (BO) or apical out (ApO) configuration in expansion (exp) medium or differentiation (diff) medium: markers of a basal (P63) cells (A), goblet (MUC5AC) cells (B), club (SCGB1A1) cells (C), and ciliated (FOXJ1) cells (D). Data shown as mean ± SD of a triplicate experiment, * denotes p < 0.05, ** denotes p < 0.01 for ANOVA with post-hoc statistical testing.

## Supplementary Figure 2


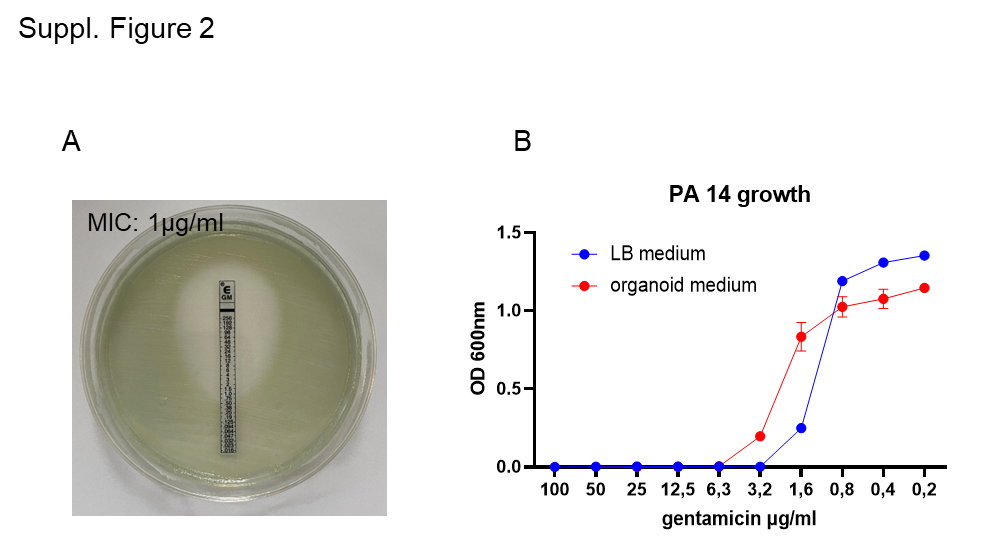


**Supplementary Figure 2.** Determination of the minimal inhibitory concentration (MIC) of gentamicin in *Pseudomonas aeruginosa* (PA) with conventional microbiological methods (Etest, MIC: 1µg/ml (A)) and in experiment-specific conditions (growth in ALI or LB medium; B). This allowed us to minimize the use of gentamicin in our infection model (8µg/ml) and thus minimize off-target effects.

## Supplementary Figure 3


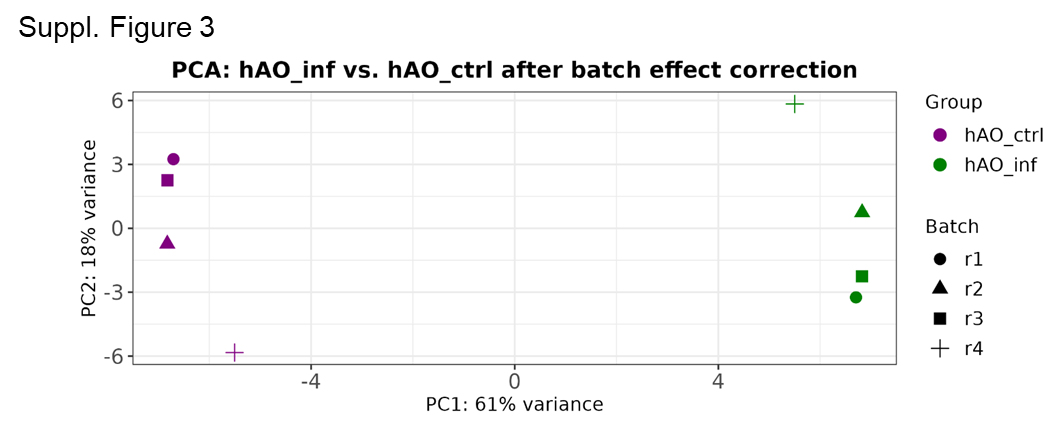


**Supplementary Figure 3.** Principal component analysis (PCA) after batch effect correction of control AOs (hAO_ctrl) or AOs infected with *Pseudomonas aeruginosa* (hAO_inf).

# Supplementary Tables

## Supplementary Table 1

| Target | Forward primer | Reverse primer |
| --- | --- | --- |
| TUB | TCCTTCAACACCTTCTTCAGTGAGACG | GGTGCCAGTGCGAACTTCATCA |
| OAZ1 | GGATCCTCAATAGCCACTGC | TACAGCAGTGGAGGGAGACC |
| IL-6 | AGCCCACCGGGAACGAAAGAGA | AAGGCAGCAGGCAACACCAGG |
| IL-8 | AGCCTTCCTGATTTCTGCAG | GTCCACTCTCAATCACTCTCA |
| NOS2 | GTTTGACCAGAGGACCCAG | ATCTCCTTTGTTACCGCTTCC |
| 16s | CAA AACTACTGAGCTAGAGTACG | TAAGATCTCAAGGATCCCAACGGC |
| RpoD | GGG CGAAGAAGGAAATGGTC | CAGGTGGCGTAGGTGGAGAA |
| NirS | ATC GAC AAC CTC ACC GTC AC | CGGCAACCTTGTTGGAGTTG |
| fhp | CAATGGCGTGCTGATGTACG | CAAGTGCCGACGATCGGATA |
| NosZ | GTTGATCGACATTTCCGGCG | CTTGCGGGTCTTGATCTGGT |
| NorB | ACCCGCGACAAGTTCTACTG | GCACCCATGATCAGTTCCCA |

Supplementary Table 1. TaqMan PCR primers used in this study

## Supplementary Table 2

[Suppl. Table 2 is provided as an Excel sheet]

Supplementary Table 2. Significantly regulated genes of AOs infected with *Pseudomonas aeruginosa* (hAO_inf) versus control AOs (hAO_ctrl) after 4h of gentamicin protected infection.

# References

1. Sachs N, Papaspyropoulos A, Zomer-van Ommen DD, Heo I, Böttinger L, Klay D, et al. Long-Term Expanding Human Airway Organoids for Disease Modeling. *EMBO J* (2019) 38(4). Epub 2019/01/16. doi: 10.15252/embj.2018100300.

2. Co JY, Margalef-Català M, Monack DM, Amieva MR. Controlling the Polarity of Human Gastrointestinal Organoids to Investigate Epithelial Biology and Infectious Diseases. *Nature Protocols* (2021) 16(11):5171-92. doi: 10.1038/s41596-021-00607-0.

3. Zhou J, Li C, Sachs N, Chiu MC, Wong BH, Chu H, et al. Differentiated Human Airway Organoids to Assess Infectivity of Emerging Influenza Virus. *Proc Natl Acad Sci U S A* (2018) 115(26):6822-7. Epub 2018/06/13. doi: 10.1073/pnas.1806308115.

4. Grace A, Sahu R, Owen DR, Dennis VA. Pseudomonas Aeruginosa Reference Strains Pao1 and Pa14: A Genomic, Phenotypic, and Therapeutic Review. *Front Microbiol* (2022) 13:1023523. Epub 2022/11/01. doi: 10.3389/fmicb.2022.1023523.
